# Stabilizing Prefusion SARS-CoV-2 Spike by Destabilizing the Postfusion Conformation

**DOI:** 10.3390/vaccines13030315

**Published:** 2025-03-14

**Authors:** Debajyoti Chakraborty, Randhir Singh, Raju S. Rajmani, Sahil Kumar, Rajesh P. Ringe, Raghavan Varadarajan

**Affiliations:** 1 Molecular Biophysics Unit, Indian Institute of Science, Bangalore 560012, India; debajyotic@iisc.ac.in (D.C.); raju.rajmani@yahoo.in (R.S.R.); 2 Mynvax Private Limited, 3rd Floor, Brigade MLR Centre, No.50, Vani Vilas Road, Basavanagudi, Bengaluru 560004, India; randhir.singh@mynvax.com; 3 Virology Unit, Institute of Microbial Technology, Council of Scientific and Industrial Research (CSIR), Chandigarh 160036, India; sahilkoundal999@gmail.com (S.K.); rajeshringe@gmail.com (R.P.R.)

**Keywords:** stabilization, SARS-CoV-2 Spike, charged amino acids, HR1 and HR2, immunogenicity, COVID-19

## Abstract

**Background/Objectives:** As with many viral fusion proteins, the native conformation of SARS-CoV-2 Spike is metastable. Most COVID-19 vaccines utilize a stabilized Spike (Spike-2P) containing two proline substitutions, and subsequently, a further stabilized variant with four additional proline substitutions, Spike-6P, has been developed. In an alternative approach, we introduced two aspartic acid residues (2D) in the HR1 region of Spike at positions that are exposed and buried in the pre- and postfusion states, respectively, to destabilize the postfusion conformation. **Methods:** The recombinant protein constructs were expressed in a mammalian cell culture and characterized for their yield and antigenicity, and the formulations were then used to immunize hamsters. After two immunizations, the hamsters were challenged with live B.1.351 SARS-CoV-2 virus for an evaluation of the protective efficacy. **Results:** The introduction of the two aspartic acid mutations resulted in an approximately six-fold increase in expression, comparable to that in Spike-2P. When the 2D mutations were combined with the above four proline mutations (Spike-4P-2D), this led to a further three- to four-fold enhancement of protein expression, similar to that seen in Spike-6P. When formulated with the oil-in-water emulsion adjuvant Sepivac SWE, the 2P, 2D, 6P, and 4P-2D Spike variants all protected female hamsters against heterologous challenge with the B.1.351 SARS-CoV-2 virus and elicited high titers of neutralizing antibodies. **Conclusions:** We suggest that destabilization of the postfusion conformation through the introduction of charged amino acids at sites that are exposed in the pre- and buried in the postfusion conformation offers a general strategy to enhance the yield and stability of the native, prefusion conformation of viral surface proteins.

## 1. Introduction

SARS-CoV-2, the causative agent of the COVID-19 pandemic, belongs to the family Coronoviridae and is a positive-sense, single-stranded RNA virus. It contains four major structural proteins, Spike, Envelope, Membrane, and Nucleocapsid. Spike glycoprotein facilitates the infection of SARS-CoV-2 by binding with the help of the receptor-binding domain (RBD) to human angiotensin-converting enzyme 2 (h-ACE2). Spike then undergoes several conformational changes to expose the fusion peptide, ultimately leading to fusion of the host and viral membranes [1,2,3]. Spike glycoprotein is the primary target of the host humoral response and thus important for vaccine development. Most neutralizing antibodies are directed against the RBD [4,5,6,7,8,9,10,11,12,13,14]. A variety of vaccine platforms have been used to combat COVID-19, including viral vectors, messenger RNA lipid nanoparticles (mRNA-LNPs), inactivated virus, and protein subunit approaches [15,16,17,18,19,20]. With the exception of the chimpanzee adenovirus viral vectored vaccine [21,22,23] which used the wildtype Spike, all other clinically approved Spike-based platforms have employed a stabilized version of Spike containing at least two proline mutations (Spike-2P) as the sole immunogen, as described below [24]. The 2P mutations are patent protected, and for this and other reasons, it is of interest to explore alternative approaches to stabilize Spike which do not require these mutations.

A significant challenge in viral vaccine development for enveloped viruses is the stabilization of the relevant surface protein in its prefusion, native conformation on the viral surface [25,26,27,28,29,30,31,32]. Spike and other viral fusion proteins on enveloped viruses are metastable. During the process of viral fusion, they adopt a more stable postfusion conformation, and the energy released drives the fusion process. Prefusion stabilization not only preserves neutralizing epitopes but can also enhance protein yield and stability in vitro and in vivo, thereby enhancing both immunogenicity and protective efficacy. Prefusion stabilization of Spike has been achieved using various strategies. Among them, one of the most widely used is the introduction of proline substitutions at K986 and V987 in Spike, known as the 2P mutations. These restrict the conformational flexibility of Spike and thus inhibit the transition to the postfusion conformation [24,33]. Several other prefusion stabilization methods have been used, including the introduction of four additional Pro mutations into Spike-2P to yield Spike-6P, locking specific regions of Spike using disulfide bonds, and covalent cross-linking methods, cavity-filling mutations, deletion of a fusion peptide, and Gly substitutions [24,34,35,36,37,38,39].

In the present work, in an alternative approach, we introduced two aspartic acid residues (2D) into the HR1 region of Spike at positions that are exposed and buried in the pre- and postfusion states, respectively, to destabilize the postfusion six-helix bundle conformation. Since the stability of the prefusion relative to the postfusion state equates to the difference in free energy between pre- and postfusion conformations, destabilization of the postfusion conformation will enhance the stability of the prefusion state. We have previously used a similar approach to destabilize the postfusion conformations of influenza hemagglutinin and HIV-1 Env protein. In the case of HIV-1 Env, the introduction of aspartic acid mutations prevented the sCD4-induced gp120 shedding, increased binding to neutralizing antibodies such as PG9 and PG16, and reduced the exposure of non-neutralizing epitopes, whereas in the case of the influenza hemagglutinin stem, stabilization was confirmed by assessing protein structural integrity and the antigenicity of WT and mutant proteins [40,41]. The introduction of a negatively charged Asp amino acid (D in single-letter amino acid code) at such positions should destabilize the postfusion state, and hence we hypothesized that this would enhance the yield of Spike ectodomains. We also evaluated the immunogenicity and protective efficacy of these stabilized constructs in the hamster model, as these are the model organisms which most closely replicate the pathological features found in the lungs of COVID-19 patients, and show that they provide a viable alternative to the widely used 2P mutations. The positions chosen for Asp introduction are conserved in other sarbecoviruses, suggesting the broad applicability of this strategy to enhance the yield and immunogenicity in pan sarbecovirus vaccines.

## 2. Materials and Methods

### 2.1. Identification of Mutants and Designing Constructs

The accessible surface areas for the prefusion and postfusion conformations of Spike (PDB ID: 6VXX and PDB ID: 6XRA) were analyzed using the program NACCESS, which calculates the solvent-accessible surface area (SASA) of proteins using a spherical probe (default 1.4 Å, water-sized), based on the algorithm by Lee and Richards (1971) [42,43]. The residues were selected in the HR1 and HR2 regions, based on the criteria that they are exposed in the prefusion conformation and buried in the postfusion conformation. Two such residues, N969 and I973, found to be buried in the postfusion conformation but fully exposed in the prefusion conformation, were mutated to aspartic acids. The mutations were inserted into the Spike ectodomain-encoding gene, which was cloned into a mammalian expression vector, pCDNA3.4., with a trimerization domain and a 10X histidine tag at the C-terminus. The base sequence for all the constructs was from the original Wuhan isolate (GenBank Id: YP_009724390.1).

### 2.2. Recombinant Protein Expression and Purification

The genes encoding the ectodomains of WT-Spike, Spike-2P, Spike-6P, and Spike-4P-2D were synthesized by Genscript and were cloned in the mammalian expression vector pCDNA3.4. These recombinant protein constructs were transiently transfected in Expi293F™ cells following the manufacturer’s instructions (Gibco, ThermoFisher Scientific, Waltham, MA, USA), expressed, and purified. Briefly, the Expi293F™ cells were cultured by maintaining a viable cell concentration of ~3 × 10⁶ cells/mL. For transfection, plasmid DNA and ExpiFectamine™ 293 were diluted in Opti-MEM™ I reduced serum medium and incubated at room temperature for five minutes. After an additional 15 to 20 min, the ExpiFectamine™ 293/plasmid DNA complexes were gradually added to the Expi293F™ cell culture. Eighteen hours post-transfection, ExpiFectamine™ 293 Transfection Enhancer 1 and 2 were introduced to the cells to enhance transfection efficiency. Five days after transfection, the culture was harvested, and the proteins were purified from the supernatant using nickel affinity chromatography. The supernatant was incubated with Ni-Sepharose 6 Fast Flow resin (GE Healthcare, Uppsala, Sweden) at 4 °C for 6–8 h. To eliminate non-specific proteins, the resin was washed with twenty column volumes of wash buffer (PBS containing 25 mM imidazole, pH 7.4). The proteins were then eluted from the Ni-NTA column using PBS with 500 mM imidazole (pH 7.4) and subsequently dialyzed against PBS using a dialysis membrane with a 10 kDa molecular weight cutoff. The purity of the purified proteins was confirmed through SDS-PAGE analysis. The absorbance at 280 nm for the protein was determined using the Biophotometer-D30 (Eppendorf, Hamburg, Germany). These absorbance values were then utilized to calculate the protein concentration by applying the protein-specific extinction coefficient.

### 2.3. Estimation of Protein Yields by Capture ELISA

ELISA 96-well plates were coated with the monoclonal antibody CR3022 (10 µg/mL) and were incubated at 25 °C for two hours. After incubation, the wells were blocked with 3% non-fat milk. Subsequently, purified proteins or cell supernatant containing expressed protein were added in various serial dilutions and were incubated for an hour. Following incubation, the plate was washed and mice sera raised against previously immunized Spike were added and incubated. Following incubation, the membrane was washed with PBST and incubated with an anti-mouse IgG (Fc specific)–alkaline phosphatase antibody produced in goat (Cat # A2429-Sigma-Aldrich, St. Louis, MO, USA) antibody at a dilution of 1:5000. The wells were further washed with PBST. Plates were then washed three times before the addition of alkaline phosphatase yellow (pNPP) liquid substrate (Cat# P7998, Sigma-Aldrich, USA). The reaction was incubated at 37 °C for 30 min. Absorbance was directly measured at 405 nm using a microplate reader. Protein concentrations were obtained by comparison with a standard curve obtained with purified Spike-2P.

### 2.4. Immunization of Hamsters

All hamster immunization studies received approval from the Institutional Animal Ethics Committee under approval numbers CAF/ETHICS/847/2021 and CAF/ETHICS/979/2023. The studies were conducted at the Central Animal Facility (CAF), Indian Institute of Science (IISc), in compliance with CPCSEA and ARRIVE guidelines. The animals were housed at the CAF, IISc, Bangalore, under controlled conditions with access to food and water ad libitum and a 12 h light–dark cycle. The adjuvant SWE (squalene-in-water emulsion near identical to MF59) is available as Sepivac SWE™ in GMP grade through an open-access model. The recombinant proteins were prepared by mixing with SWE in a 1:1 volume ratio while maintaining the osmolality. Female golden Syrian hamsters (*n* = 5) were used for immunization studies. Each group of hamsters was intramuscularly administered 5 μg of Spike-4P-2D, Spike-2D, Spike-2P, and Spike-6P recombinant protein formulated with SWE adjuvant on day 0 (Prime) and day 21 (Boost). Animals treated with the 1X PBS + SWE adjuvant alone served as controls. Blood samples were collected from both immunized and unimmunized animals at three time points: prior to the prime dose (Day −1), two weeks after the prime dose (Day 14), and 14 days following the booster dose (Day 35). These sera samples were analyzed for endpoint ELISA and neutralizing antibody titers.

### 2.5. ELISA for Endpoint Titer Determination

Serum-binding antibody endpoint titers were measured through ELISA. Briefly, 96-well ELISA plates were coated with 4 μg/mL of RBD (amino acids 332–532) incubated at room temperature (25 °C) for 2–3 h. The plates were washed with PBST and blocked with 3% non-fat milk. Serial four-fold dilutions of antisera raised against immunogens were added to the wells and incubated. Following incubation, the plates were washed with PBST and were incubated with a goat anti-mouse IgG secondary antibody conjugated to alkaline phosphatase for 1 h at 25 °C. The plates were washed again and incubated with pNPP substrate at 37 °C for 30 min. The optical density (OD) was read at 405 nm, and endpoint titers were determined as the highest dilution with an OD exceeding 0.2 at 405 nm.

### 2.6. Pseudoviral Neutralization Assay

Pseudoviral neutralization assays for the sera obtained from the blood collected 14 days after the boost immunization were performed using the B.1.351 Spike variant displayed on a pHIV-1 NL4.3Δenv-Luc backbone, following previously established protocols [9]. Neutralization titers (ID50) were calculated as the serum dilution required to achieve 50% inhibition of viral infectivity.

### 2.7. Challenge Studies of Hamsters

The virus challenge study was conducted following established protocols. Following immunization, the hamsters were moved to the BSL-3 virus facility at the Centre for Infectious Disease Research, Indian Institute of Science, Bangalore, India. They were housed in individually ventilated cages (IVCs) under controlled conditions (23 ± 1 °C and 50 ± 5% relative humidity). After acclimatization for a period of 7 days, the hamsters were intranasally challenged with 105 PFU of SARS-CoV-2 B.1.351 live virus (obtained from BEI Resources) in 100 μL of DMEM. Anesthesia, containing a cocktail of xylazine (10 mg/kg body weight) and ketamine (150 mg/kg body weight), was administered intraperitoneally prior to the challenge of the hamsters. Health parameters, including body weight, temperature, and clinical signs, were monitored daily by a veterinarian.

### 2.8. Histopathological Examinations

Six days post-challenge, the hamsters were humanely euthanized via an overdose of xylazine administered intraperitoneally and lung tissues were collected. We processed 4% paraformaldehyde-fixed lungs, which were embedded in paraffin and cut into 4 um sections by a microtome for hematoxylin and eosin staining. The lung sections were microscopically examined and evaluated for different pathological scores. For lung tissue histopathology scoring, we developed a scientific method using Mitchison’s virulence scoring system with some modifications, considering the consolidation of lungs, severity of bronchial and alveolar inflammation, immune cell influx, and alveolar and perivascular edema [9,44]. The histopathology scores were graded as 0–4 (4: Severe pathology; 3: Moderate pathology; 2: Mild pathology; 1: Minor/minimum pathology; 0: No pathology).

### 2.9. Statistical Analysis

Data analysis was performed using GraphPad Prism software version 10. The ELISA binding, neutralization titers, and pseudoviral virus neutralization titer data were analyzed with a two-tailed Mann–Whitney test and non-parametric Kruskal–Wallis test with Dunn’s multiple comparisons, respectively. Weight changes in hamsters were analyzed with a two-tailed Student’s *t* test (* indicates *p*  <  0.05, ** indicates *p*  <  0.01, *** indicates *p*  <  0.001, **** indicates *p*  <  0.0001).

## 3. Results

### 3.1. Identification of Mutations to Destabilize the Prefusion Conformation of the Full-Length Spike Protein

**Figure 1:**
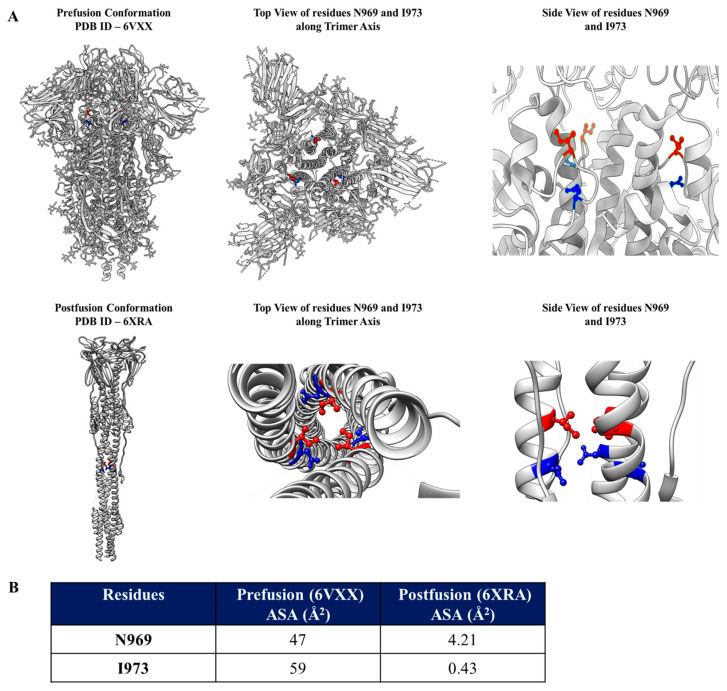
Identification of residues to be mutated to Asp (D) to stabilize Spike in its prefusion conformation. (**A**). Locations of residues N969 (Blue) and I973 (Red) in the prefusion (**top** panel) and in the postfusion (**bottom** panel) structures. In the adjoining panels, the residues are depicted in enlarged views (top view along the trimeric axis and side view, 90 degree from the top view) to show that they are exposed and buried in the prefusion and postfusion states, respectively (**B**). Accessible surface areas of the two selected residues in their prefusion and postfusion states.

**Figure 2:**
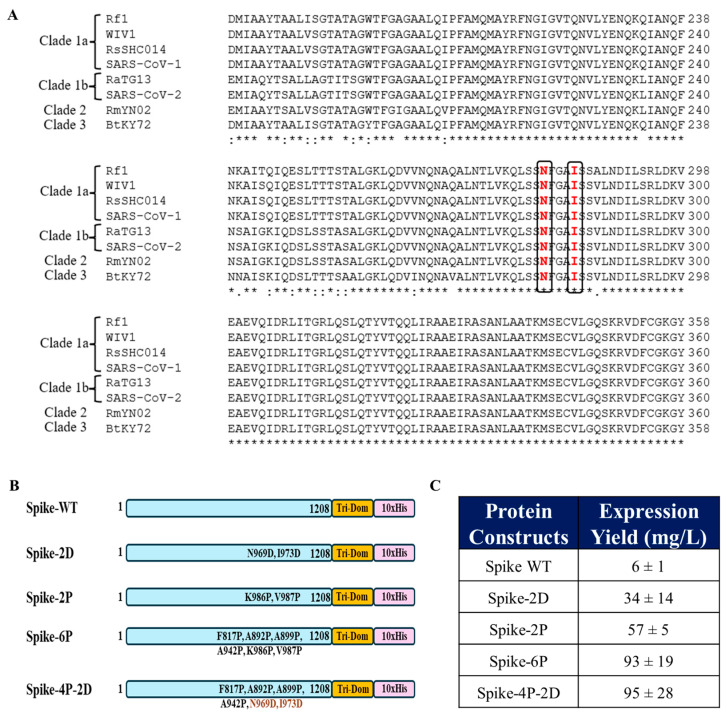
Conservation of N969 and I973 in Spikes across sarbecovirus clades and yields of different stabilized SARS-CoV-2 Spikes. (**A**). Multiple sequence alignment of the S2 region of Spike across various clades of sarbecoviruses. Conserved residues N969 and I973 are highlighted. (**B**). Different Spike constructs made to compare and evaluate the prefusion stabilization by measuring protein yields, immunogenicity, and protective efficacy (Tri-Dom—trimerization domain, 10XHis—10X histidine tag). (**C**). A comparison of the purified yields (mg/L) of various stabilized Spike proteins transiently expressed in Expi293 mammalian cells (*n* = 3). In the case of Spike WT, pure protein could not be isolated due to inadequate expression; therefore, the yield was estimated by ELISA using purified Spike-2P as a standard.

To identify the mutations which can destabilize the prefusion conformation of SARS-CoV-2 Spike, accessibility calculations were performed individually for the prefusion and postfusion conformations of Spike (6VXX and 6XRA). Residues were screened based on whether they were exposed in the prefusion and become buried in the postfusion conformation (Figure 1A,B). Two residues, N969 and I973, were found to be buried in the postfusion conformation but fully exposed in the prefusion conformation. The identified residues were substituted by aspartic acid in the mutants N969D and I973D, to destabilize the postfusion conformation, as discussed above. Interestingly, residue N969 is mutated to lysine (K) in recent variants of concern, Omicron XBB.1.5, JN.1, KP.2, and BA.5. Both residues, N969 and I973, are conserved in Spikes across various clades of sarbecoviruses (Figure 2A).

### 3.2. Introduction of the N969D and I973D Mutations into the Ectodomain of the WT Spike Increases Expression and Obviates the Need for the K986P and V987P (2P) Mutations

The SARS-CoV-2 Spike-WT ectodomain as well as the Spike ectodomain containing the N969D and I973D mutations (referred to as Spike-2D) with a trimerization domain attached to the C-terminus were transiently transfected and expressed in Expi293 mammalian cells. Simultaneously, the ectodomain of Spike-2P, with the above two proline mutations, the ectodomain of Hexapro Spike (Spike-6P), with four additional proline mutations at F817P, A892P, A899P, and A942P with the C-terminal trimerization domain, were transiently transfected and expressed in Expi293 cells. Additionally, transfection in Expi293 cells was carried out for a construct (Spike-4P-2D) produced by reverting the K986P and V987P mutations in Spike-6P and introducing the N969D and I973D mutations in this ectodomain (Figure 2B). The recombinant proteins were purified using nickel affinity chromatography. The introduction of the aspartic acid mutations, Spike-2D, increased the yield of Spike ectodomain substantially in comparison to WT Spike. The latter could not be purified in sufficient amounts for accurate yield estimation. The yields of Spike-2P and Spike-2D were comparable. The Spike-4P-2D and Spike 6P ectodomains had similar yields when expressed transiently in Expi293 cells, which were higher that of the previously reported yield for Spike-6P from transient expression in ExpiCHO cells [24] (Figure 2C). Thus, the introduction of two mutations in Spike (Spike-2D), along with an additional four proline mutations (Spike-4P-2D), stabilized the Spikes in their prefusion conformation, as assayed by CR3022 neutralizing antibody binding, and resulted in a significant increase in the protein yields of the Spike ectodomain, which eliminates the need for K986P and V987P mutations.

### 3.3. Spike-4P-2D Elicited a Potent Neutralizing Antibody Response in Hamsters

**Figure 3:**
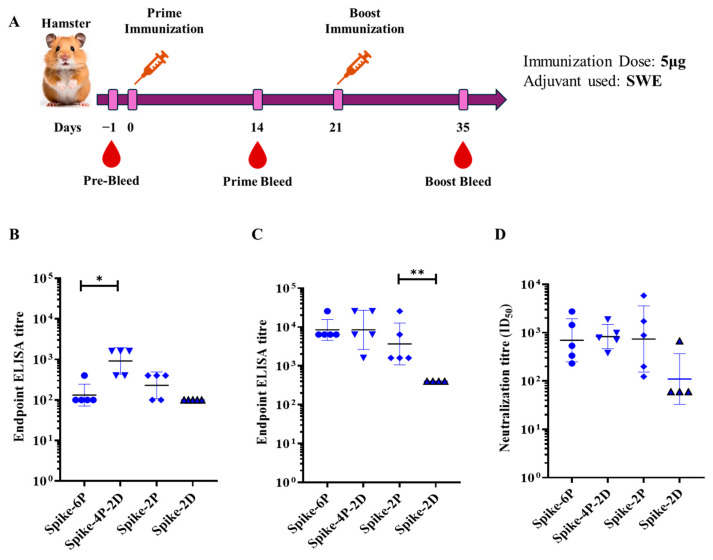
ELSA and neutralizing antibody titers elicited following immunization of various stabilized SARS-CoV-2 Spike derivatives. (**A**). Immunization regimen of hamsters. Female Syrian hamsters were immunized twice with 5 µg of different prefusion stabilized constructs of Spikes. (**B**). ELISA endpoint titers against the receptor-binding domain after prime immunization. (**C**). ELISA endpoint titers against the receptor-binding domain after the boost immunization. (**D**). Neutralization titers against the B.1.351 pseudoviruses for the sera obtained from the blood collected post 14 days after the boost immunization. The endpoint ELISA binding titers and the pseudoviral neutralization titers were analyzed with a two-tailed Mann–Whitney test (* indicates *p*  <  0.05, ** indicates *p*  <  0.01).

Female Syrian golden hamsters were immunized twice with 5 μg of Spike-4P-2D, Spike-2D, Spike-2P, and Spike-6P, with SWE as an adjuvant. Prime immunization was administered on day 0, whereas the boost immunization was administered on day 21 (Figure 3A). A control group of hamsters was administered the SWE adjuvant with PBS. ELISA was performed to evaluate the endpoint antibody titers elicited by the immunized Spikes. All the Spike constructs elicited significant antibody titers after a prime immunization (Figure 3B). The endpoint ELISA titers after a single boost immunization for Spike-4P-2D were comparable to those of Spike-6P and Spike-2P (Figure 3C). All sera from the boost immunizations neutralized the heterologous B.1.351 pseudovirus (Figure 3D).

### 3.4. All Spike Derivatives Confer Protection Against a Heterologous SARS-CoV-2 (B.1.351) Live Virus Challenge in Hamsters

**Figure 4:**
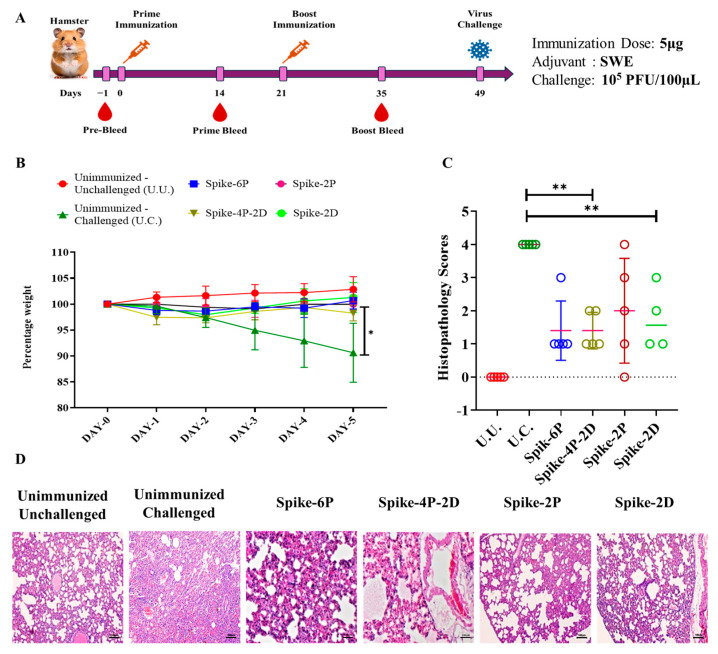
Protective efficacy of prefusion stabilized Spike constructs against a heterologous live virus challenge. (**A**). Immunization and challenge study regimen. Female Syrian hamsters were immunized twice with 5 µg of different prefusion stabilized constructs of Spikes and were then challenged with 10^5^ PFU/100 µL of B.1.351 SARS-CoV-2 virus. (**B**). Average weight change in hamsters up to five days post B.1.351 virus challenge. All immunized hamsters recovered from the challenge, whereas the unimmunized challenged (U.C.) did not. (**C**). Histopathology scores of lungs. (**D**). Histopathology of lungs of unimmunized unchallenged (U.U.), unimmunized challenged (U.C.), immunized Spike-6P, Spike-4P-2D, Spike-2D, and Spike-2P. Clear and well-defined epithelial interstitial spaces and decreased immune cell infiltration can be observed in immunized challenged hamsters in comparison to unimmunized challenged hamsters. All images are shown at the same magnification. Scale bar: 100 µm. Weight changes were analyzed with a multiple Student’s *t* test and the Bonferroni Dunn correction method (* indicates *p*  <  0.05, ** indicates *p*  <  0.01).

To evaluate the protective efficacy of Spike-4P-2D against live virus challenge, female Syrian golden hamsters were immunized with 5 μg of Spike-4P-2D, Spike-2D, Spike-2P, and Spike-6P proteins formulated with SWE adjuvant and were challenged with 1 × 10^5^ PFU of live B.1.351 (Beta) virus 28 days post boost immunizations (Figure 4A). One control group consisted of hamsters immunized with SWE plus PBS, which was challenged with virus, while the other mock immunized group was unchallenged. All immunized hamsters were protected from live virus challenge (Figure 4B). Evaluation of the lung tissue sections from immunized hamsters showed well-defined epithelial interstitial spaces and decreased immune cell infiltration compared to the B.1.351 challenged group without immunization (Figure 4C).

## 4. Discussion

Vaccines played a critical role in controlling the COVID-19 pandemic. The structural integrity and conformation of the immunogens are important factors to be considered while designing a vaccine candidate. Stabilization of Spike protein in its native form is helpful for enhanced expression and for eliciting neutralizing antibodies. Introducing proline substitutions, K986 and V987, in the S2 subunit of Spike protein is one of the most widely used stabilization approaches, which was initially demonstrated in Middle East respiratory syndrome coronavirus (MERS-CoV) and later employed for SARS-CoV-2 [33]. As discussed, the stabilization of Spike protein has a significant impact on the expression yield and hence the vaccine efficacy in both mRNA and protein subunit vaccine platforms. The mRNA vaccines developed by Pfizer-BioNTech (BNT162b2) and Moderna (mRNA-1273) utilized 2P-stabilized Spike. These vaccines were crucial in combating the COVID-19 pandemic by eliciting both strong neutralizing antibody responses and cellular immunity. These stabilizing substitutions lock Spike protein in its prefusion state and thus prevent premature conformational changes, possibly through decreased conformational entropy of the unfolded state. A similar strategy was used to further stabilize the prefusion conformation of Spike protein by introducing four extra proline residues in addition to the 2P mutations (HexaPro), which enhanced thermal stability and protein yield [37,38,39].

In this study, we employed aspartic acid substitutions to stabilize SARS-CoV-2 Spike in its prefusion conformation. The introduction of aspartic acid mutations into WT Spike significantly increased the yields of the protein while retaining conformational integrity and obviated the need for the K986P and V987P (2P) mutations. The recombinant Spike proteins, when formulated with SWE adjuvant and immunized, were found to induce significant neutralizing antibody titers in hamsters. The boost sera obtained from the animals could neutralize the B.1.351 pseudovirus. The protective efficacy of the recombinant Spike constructs was also evaluated using a live viral challenge study. The immunized hamsters were challenged with a lethal dose of live B.1.351 SARS-CoV-2 virus. The Spike 4P-2D and Spike 2D constructs conferred equivalent protection to Spike-6P and Spike-2P. All immunized hamsters showed reduced lung pathology compared to unimmunized controls.

As the SARS-CoV-2 virus continues to evolve in response to immune pressure, resulting in newer variants, research into Spike protein stabilization strategies continues to be important for next-generation coronavirus vaccines. Enhanced stability could improve vaccine durability and elicit cross-protection against emerging variants as well as enhance pandemic preparedness.

## 5. Conclusions

In conclusion, our study highlights the utility of such charged amino acid substitutions to enhance prefusion stability and increase the yield and immunogenicity of SARS-CoV-2 Spike protein. The conservation of these residues (Figure 2A) suggests that the same mutations can be used to stabilize Spike proteins from other sarbecoviruses. We have previously used a similar approach to stabilize the prefusion conformations of influenza hemagglutinin and HIV-1 Env protein [40,41], suggesting that destabilization of the postfusion conformation through the introduction of charged amino acids at buried sites offers a general strategy to enhance the yield and stability of the native, prefusion conformation of viral surface proteins.

## 6. Patents

A provisional patent application has been filed for the 2D mutations, N969D and I973D, described in this manuscript. R.V. and R.S. are inventors on that application.

## Data Availability

All data are available in the main text.

## Abbreviations

The following abbreviations are used in this manuscript:

RBD	Receptor-Binding Domain
SARS-CoV-2	Severe Acute Respiratory Syndrome Coronavirus 2
SWE Adjuvant	Squalene-in-Water Emulsion Adjuvant
